# Research into the effect Of SGLT2 inhibition on left ventricular remodelling in patients with heart failure and diabetes mellitus (REFORM) trial rationale and design

**DOI:** 10.1186/s12933-016-0419-0

**Published:** 2016-07-15

**Authors:** Jagdeep S. S. Singh, Amir Fathi, Keeran Vickneson, Ify Mordi, Mohapradeep Mohan, J. Graeme Houston, Ewan R. Pearson, Allan D. Struthers, Chim C. Lang

**Affiliations:** Division of Molecular and Clinical Medicine, Ninewells Hospital and Medical School, University of Dundee, Dundee, UK

**Keywords:** Heart failure, Diabetes, SGLT2 inhibitor, Mechanistic trial, Cardiac MRI, Cardiopulmonary exercise testing

## Abstract

**Background:**

Heart failure (HF) and diabetes (DM) are a lethal combination. The current armamentarium of anti-diabetic agents has been shown to be less efficacious and sometimes even harmful in diabetic patients with concomitant cardiovascular disease, especially HF. Sodium glucose linked co-transporter type 2 (SGLT2) inhibitors are a new class of anti-diabetic agent that has shown potentially beneficial cardiovascular effects such as pre-load and after load reduction through osmotic diuresis, blood pressure reduction, reduced arterial stiffness and weight loss. This has been supported by the recently published EMPA-REG trial which showed a striking 38 and 35 % reduction in cardiovascular death and HF hospitalisation respectively.

**Methods:**

The REFORM trial is a novel, phase IV randomised, double blind, placebo controlled clinical trial that has been ongoing since March 2015. It is designed specifically to test the safety and efficacy of the SLGT2 inhibitor, dapagliflozin, on diabetic patients with known HF. We utilise cardiac-MRI, cardio-pulmonary exercise testing, body composition analysis and other tests to quantify the cardiovascular and systemic effects of dapagliflozin 10 mg once daily against standard of care over a 1 year observation period. The primary outcome is to detect the change in left ventricular (LV) end systolic and LV end diastolic volumes. The secondary outcome measures include LV ejection fraction, LV mass index, exercise tolerance, fluid status, quality of life measures and others.

**Conclusions:**

This trial will be able to determine if SGLT2 inhibitor therapy produces potentially beneficial effects in patients with DM and HF, thereby replacing current medications as the drug of choice when treating patients with both DM and HF.

*Trial registration* Clinical Trials.gov: NCT02397421. Registered 12th March 2015

## Background

Heart failure (HF) is a growing global pandemic with a 14 % increase in prevalence from 1990 to a staggering 41 million patients worldwide in 2010 [1]. With improving acute cardiovascular outcomes and longer life expectancy, this number will rise further. HF is already one of the leading causes of hospitalisation in elderly patients [2]. This translates to a burgeoning financial burden on the healthcare system; costing $39.2 billion in the US alone in 2010 [3].

HF is associated with numerous co-morbidities that can contribute to the progression of the disease and may alter the response to therapy [4]. One important co-morbidity of HF is diabetes mellitus (DM). In population based studies and in HF trials, the prevalence of type 2 DM among patients with symptomatic HF is estimated to be between 12 and 49 % [5, 6]. Among all patients hospitalized for HF, it has been reported that up to 40 % have type 2 DM [7, 8]. This association can be lethal since DM has consistently been shown to be an independent predictor of increased morbidity and mortality in patients with HF; [9] patients with DM and HF have a median survival of 4 years [10]. However, treating patients with these concomitant diseases can be challenging.

### Treating heart failure and diabetes

In the treatment of DM, EASD/ADA guidelines recommend tailoring therapeutic approaches to individual needs and/or risks [11]. For most patients, metformin is the first choice anti-diabetic drug in type 2 DM including those with HF. In 2010, investigators reported potential benefits of metformin therapy in HF in DM in the Scottish population [12], a finding that has since been confirmed by others [13, 14]. However, metformin alone is often not enough to keep glycaemia under control and there frequently is a need for a second line anti-diabetic drug in patients with HF and DM. However, the choices for patients with concomitant HF are very limited; sulphonylureas (SU) are agents that are commonly prescribed in DM but are associated with weight gain and hypoglycaemia which are detrimental in heart failure [15, 16]. Moreover, there remain concerns that SUs may increase all-cause and CV-mortality [17], although this link is not fully established. Glitazones are contra-indicated in New York Heart Association functional class (NYHA) III or IV HF, while their role in milder degrees of HF remain somewhat controversial with a few observational studies indicating increased HF hospitalisation [18]. More recent agents such as DPPIV inhibitors have also failed to show cardiovascular benefit; TECOS showed sitagliptin had a neutral effect on cardiovascular outcomes [19]. Similarly EXAMINE revealed alogliptin had no effect on major adverse CV events (MACE) among diabetic patients with recent ACS [20]. While SAVOR-TIMI-53 revealed that saxagliptin increased HF hospitalisations [21]. Therefore, we can conclude that second line therapeutic options in DM and HF are very limited and there is a critical need for agents that will both improve glycaemic control as well as HF outcomes.

### Sodium glucose linked co-transporter 2 (SLGT2) inhibitors and the heart

**Fig. 1:**
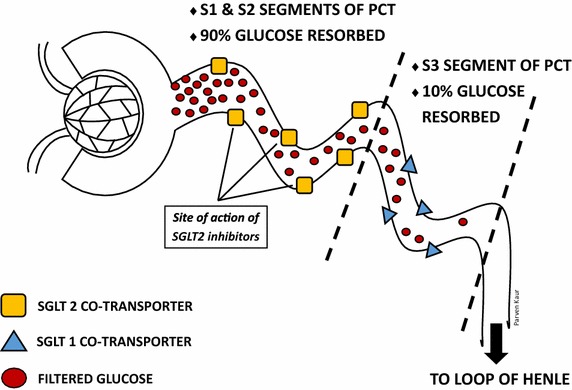
Normal renal tubular resorption of glucose. The figure above depicts the physiological resorption of glucose in the PCT of the nephron. SGLT2 co-transporters located at the S1 and S2 segments of the PCT are responsible for 90 % of the resorbed glucose, whereas SGLT1 co-transporters remove the remainder in the S3 segment. The diagram also identifies the site at which SGLT2 inhibitors act. *PCT* proximal convoluted tubules

SGLT2 inhibitors employ a novel mechanism to lower blood glucose by preventing the reabsorption of glucose in the renal tubules. There are currently 3 agents that have been licenced for clinical use; dapagliflozin, empagliflozin and canagliflozin. By competitively blocking the SGLT2 receptors in the proximal convoluted tubules (PCT), SGLT2 inhibitors prevent the reabsorption of filtered sodium and glucose, resulting in glycosuria and natriuresis [22, 23] (Fig. 1). This novel mechanism of action means SGLT2 inhibitors function independently of insulin levels, pancreatic function and degree of insulin resistance. Accordingly, this class of drug is expected to continue to maintain its potency as beta cell function deteriorate along with disease progression—a key feature that is not seen in other oral anti-diabetic drug classes [24]. Another distinctive feature of SGLT2 inhibitors is its low hypoglycaemic risk. By limiting its activity to urinary glucose excretion, SGLT2 inhibitors do not stimulate insulin release [25] or interfere with the physiologic response to hypoglycaemia [26].

The additional natriuretic effect (and resultant osmotic diuresis) of SGLT2 inhibitors could potentially be beneficial in patients with cardiovascular disease, especially those with HF, thereby distinguishing SGLT2-inhibitors from all the other oral anti-diabetic agents. Indeed SGLT2-ihibitors have been shown to have a number of positive cardiovascular effects on top of their glycaemic effects. This class of drug has been shown to lower blood pressure (by 7–10 mmHg) [27, 28], reduce arterial stiffness [29], reduce urinary microalbuminuria [30] (a marker of CV risk) and reduce triglycerides and increase HDL and LDL cholesterol (without altering HDL/LDL ratios) [24].

Recently, the EMPA-REG Outcomes trial had demonstrated a remarkable reduction in CV mortality and HF hospitalisations, by 38 and 35 % respectively, among patients with high CV risk who were treated with empagliflozin [31]. Further analysis of the data suggested that this benefit was consistent in patients with or without HF at baseline [32]. However, it is important to note that EMPA-REG Outcomes studied a broad range of CV risk patients and only 10 % had HF at baseline, thus raising the possibility the outcomes seen in this group be due to chance. Nevertheless, such striking results warrants further inquiry. Interestingly, separation of the event curves in EMPA-REG outcomes were seen very early—within 3 months—leading some to speculate that the osmotic diuresis effect of SGLT2-inhibitors was responsible for this, as its effect other mechanisms such as LV remodelling and atherosclerosis would have taken longer to manifest. However, there has yet to be a mechanistic trial to test this hypothesis. As we specify below, the REFORM Trial will rigorously test the mechanisms behind the potential cardiovascular benefits of the SGLT2-inhibitor, dapagliflozin, specifically in the diabetic heart failure population.

## Methods

### Study design

**Fig. 2:**
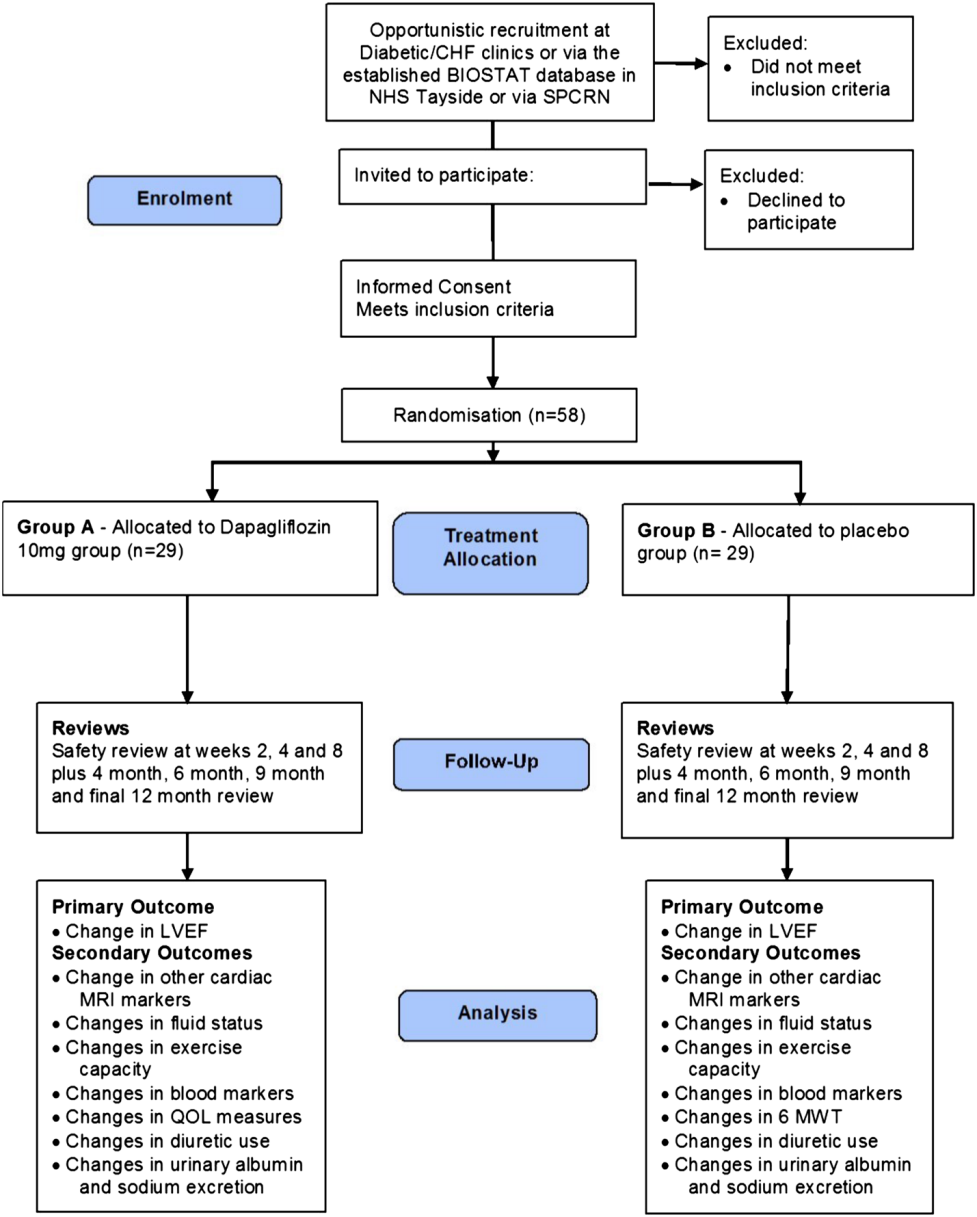
Study design flowchart. *HF* Heart failure; *LVESV* Left ventricular end systolic volume; *LVEDV* Left ventricular end diastolic volume; *LV* Left ventricular; *LVEF* Left ventricular ejection fraction; *MRI* Magnetic resonance imaging; *QOL* Quality of life

The REFORM trial is a randomised, double blinded, placebo controlled single-centre study conducted in NHS tayside, Scotland to compare the SGLT2 inhibitor, dapagliflozin 10 mg to placebo (standard of care). A recruitment window of 1.5 years has been set between March 2015 and August 2016. Participants will be enrolled in this trial for a period of between 12 to 13 months, (Fig. 2) therefore the overall trial end date will be August 2017.

At the screening visit an initial medical history and clinical examination will be performed following informed consent. Participants will have bloods taken for safety analysis and vital signs recorded to confirm eligibility prior to enrolment. Should the participant meet the inclusion criteria and have no exclusions identified they will return for a cardiac magnetic resonance imaging (MRI) scan at the Clinical Research Centre, Ninewells Hospital, Dundee, within 4 weeks of the planned baseline (randomization) visit. At the randomization visit participants will complete a 6 min walk test, quality of life measures, vital signs, body composition analysis (BCA) and cardio-pulmonary exercise test (CPEX) measurements taken. During this visit, participants will also be randomly assigned to either Dapagliflozin 10 mg or matching placebo. The first dose will be administered at this visit and participants will be educated on the symptoms of hypoglycaemia and given a written action plan on how to manage it in the event it occurs. Patients on insulin will have their total daily dose reduced by 10 % and given a 2-week glucose monitoring chart with written instructions to self-manage their insulin doses. This will be reviewed by the research team at the next visit.

Participants will return 2 weeks later for a short safety visit where they will have safety and BNP bloods, insulin dose (if applicable), adverse events and vital signs monitored. This will be followed by 2 more visits on months 2 and 6 with the same agenda. Three telephone visits are planned in the study schedule, at week 4, months 4 and 9. These calls will enable the research team to follow up on changes in concomitant medications, adverse events and to remind the participant of study drug compliance. Participants will continue with all their usual medications, these remain unchanged throughout unless clinically indicated. If any titration of a participant’s other medications (i.e. anti-diabetic agents or diuretic agents) is indicated these changes will be done, in consultation with their general practitioners (GP), and the changes recorded for analysis later.

At the end of the 1 year study period, participants will return for a repeat assessment of the 6 min walk test, quality of life measures, BCA, CPEX and cardiac MRI. These values will be compared to their baseline tests to determine if any significant change has occurred with each of the two arms of the study populations. (See Table 1 for an overview of all visits scheduled for the trial).

**Table 1:** Overview of all visits and tests scheduled for participants of the REFORM Trial

Visit	Visit 1 screening	Visit 2 baseline/randomisation	Visit 3	Visit 4 tele call	Visit 5	Visit 6 tele call	Visit 7	Visit 8 tele call	Visit 9 final visit	Early discontinuation visit^b^
	Up to 4 weeks pre visit 2	Day 0	2 week (±3 days)	4 week (±3 days)	Month 2 (±1 week)	Month 4 (±1 week)	Month 6 (±1 week)	Month 9 (±1 week)	Month 12 (±2 weeks)	As required
Informed consent	X									
Check inclusion/exclusion criteria	X									
Medical history and family history	X									
Clinical examination	X									
Demographics	X									
Vital signs	X	X	X		X		X		X	*X*
Safety blood tests and BNP	X^A^	X^A^	X^B^		X^A^		X^A^		X^A^	*X* ^*A*^
Research bloods		X							X	*X*
Genetic blood sample (if consented)		X								
MRI^a^		X							X	*X*
Bioelectrical composition analysis		X	X		X		X		X	*X*
Cardiopulmonary exercise testing		X							X	*X*
6 min walk test		X							X	*X*
QoL questionnaires		X							X	*X*
Dispense study medication		X	X		X		X			
Adverse event assessment		X	X	X	X	X	X	X	X	*X*
Record or review concomitant meds	X	X	X	X	X	X	X	X	X	*X*

Safety blood tests = X^A^ = U&Es, LFTs, FBC, glucose, HB A1C, BNP: X^B^ = U&Es, LFTs, FBC, glucose, BNP only

^a^Screening MRI to be done only if echo criteria fulfilled. Note MRI can be done (± 2 weeks of scheduled visits 2 and 9 date)

^b^Early discontinuation visit: all tests to be done only where participant agrees

^c^Urine pregnancy testing on females of childbearing potential or who do not abstain from sex or use effective contraception

### Study population

We will employ a two-pronged recruitment process. Firstly, we will identify potential patients from the local tayside pool of the systems biology study to tailored treatment in chronic heart failure (BIOSTAT) database consisting of around 1800 patients with HF who have previously consented to be approached for future research. We will also identify patients from the Scottish Primary Care Research Network (SPCRN), SHARE The Scottish Health Research Register, Generation Scotland Database, Scottish Diabetes Research Network and Wellcome Study Database. Secondly, we will also allow opportunistic recruitment from the various cardiovascular and HF clinics as well as from the cardiac rehabilitation program conducted in Ninewells Hospital. All these sources should provide a sufficient pool of diabetic HF patients to be recruited into the trial.

### Eligibility

All potential participants who meet the following inclusion and exclusion criteria will be eligible for the trial:

Inclusion criteria:

- Aged 18–75 years were previously diagnosed with type 2 DM.
- Diagnosed with NYHA functional I–III HF with prior echocardiographic evidence of left ventricular systolic dysfunction (LVSD) (ejection fraction <45 % or subjective assessment of LV dysfunction that is mild or worse).
- On furosemide 80 mg daily or less, or equivalent loop diuretic.
- Have stable HF symptoms for at least 3 months prior to consent.
- On stable therapy for HF for at least 3 months prior to consent.
- Have not been hospitalised for HF for at least 3 months prior to consent.

Exclusion criteria:

- Severe hepatic disease.
- Renal disease defined as CKD stage 3b or worse (i.e. eGFR <45 ml/min).
- Systolic BP <95 mmHg at screening visit.
- Screening HbA1c <6.0 %.
- Unable to walk or to perform cardio pulmonary exercise testing or 6MWT.
- Malignancy (receiving active treatment) or other life threatening diseases.
- Pregnant or lactating women.
- Any contraindication to MRI (e.g. claustrophobia, metal implants, penetrative eye injury or exposure to metal fragments in eye requiring medical attention).
- Patients who have participated in any other clinical trial of an investigational medicinal product within the previous 30 days.
- Patients who are unable to give informed consent.
- Any other reason considered by a study physician to be inappropriate for inclusion.

### Randomisation and treatment allocation

After successful screening for eligibility and safety, participants will be randomised to either dapagliflozin 10 mg or matching placebo (microcrystalline cellulose Ph Eur overencapsulated in a hard gelatine capsule shell) in a double blind fashion. The double blind medication (dapagliflozin or placebo) will be prepared, packaged and labelled by our onsite clinical trials pharmaceutical manufacturer. Randomisation will be carried out by our dedicated clinical trials pharmacy using block randomisation. They will use a validated randomisation program and will securely backup both the randomisation seed and the randomisation allocation and have it available in the onsite 24-h emergency unblinding facility.

Once randomised, the participant will continue taking the trial medication once daily for 1 year, if tolerated. Compliance will be checked and documented, by the dispensing pharmacy, using tablet counts at each visit. If non-compliant, they will be encouraged to become compliant. If study drug needs to be stopped due to intolerance or adverse events, they will remain in the study in order to do an “intention to treat” analysis.

### Study outcomes

#### Primary outcomes

The primary outcome is to determine if dapagliflozin induces a change in LV end systolic volume (ESV) or LV end diastolic volume (EDV) in patients with DM associated HF when compared to placebo.

#### Secondary outcomes

- To determine if there is a change in LV mass, LV ejection fraction (EF), right ventricular (RV) EDV, RV ESV, RV EF, atrial dimensions and volumes, and LV remodelling index (RI) (LV mass/LVEDV) with dapagliflozin in DM associated HF compared with placebo.
- To determine if there is a change in BCA as a measure of fluid status with dapagliflozin in DM associated HF compared with placebo.
- To determine if dapagliflozin can improve exercise tolerance in DM patients with HF as measured by pre and post 6 min walk test and CPEX.
- To determine if there are patient reported improvements in quality of life with dapagliflozin compared to placebo as measured by the Minnesota Living with Heart Failure Questionnaire and SF-36 questionnaire.
- To determine the effect of dapagliflozin on BNP, markers of inflammation and oxidative stress (IL-6, F2-isoprostane, HS-CRP and Oxidised LDL).
- To determine if dapagliflozin use results in any change in diuretic dose among patients with DM and HF.
- To determine if dapagliflozin reduces microalbuminuria in patients with DM and HF.
- To determine if dapagliflozin increases natriuresis in patients with DM and HF.
- To assess the safety of dapagliflozin in DM associated HF.

### Sample size and power calculations

Improvements in LV volumes have stood out as a marker that most strongly correlates with the impact of a drug/device therapy on improving heart failure survival [33]. Grothues et al. [34] suggests that a 10 mL change in LV EDV and LV ESV is clinically significant. In a population of HF patients the SD for the mean difference of LV EDV and LV ESV has been demonstrated to be 7.6 and 7.4 respectively [34]. Therefore, to detect a 10 mL change in EDV and ESV (primary endpoint) with 90 % power and α error (p value) of 0.05, a sample size of 13 and 12 respectively is required per arm.

LV mass is also an important determinant of survival in patients with HF [33] and a 10 g reduction in LV mass has been shown to be clinically meaningful [34]. It was also determined that the SD for 10 g mean change in LV mass in the HF population is 9.6 using cardiac MRI, implying 20 patients are required per arm [34].

The change in LV EF which best discriminated between drugs with positive and neutral effects on mortality was 3 % [35]. This is also the figure recommended by Grothues as the LV EF change which should be used to power studies [34]. Furthermore, Kramer et al. demonstrated a 3 % change in LV EF was associated with a 20 % improvement in mortality [35]. Our previous MRI experience have shown an in-house and published SD of the change in LV EF within individuals over time as 3.75 % for both active and placebo therapies [36]. Therefore in order to have 80 % power at p < 0.05 to detect a ≥3 % change in LV EF in a parallel group study, 26 patients per group are needed.

As this is a discovery trial, we aim to ensure the trial is adequately powered to detect all the clinically relevant markers of LV remodelling (LV ejection function, mass and volume). Therefore 52 patients will be recruited (26 patients per arm) to provide at least 80 % power (α error 0.05) to detect clinically significant changes in LV EDV, LV ESV, LV mass and LV EF.

### Cardiac MRI protocol

Baseline and repeat CMRI examinations in screening (±2 weeks before randomisation) and after the final 12 month (±2 weeks) visit will be performed on a 3T Magnetom scanners (Siemens, Erlangen, Germany) using body array cardiac and spine matrix radiofrequency coils. Analysis will be performed offline (Argus Software, Siemens) by a single blinded observer for the assessment of atrial and ventricular volumes and dimensions, EF, ventricular mass and LV remodelling index. This single observer will analyse all the scans.

The reproducibility of all parameters using MRI will be derived for this observer. A test–retest intra-observer coefficient of variation of 2.0 % is usual in this department’s past MRI studies. Should the scanner become unavailable for a prolonged period of time during the study an alternative scanner will be used. MRI methods will be adapted as appropriate to ensure optimal study results can be obtained

## Discussion

**Fig. 3:**
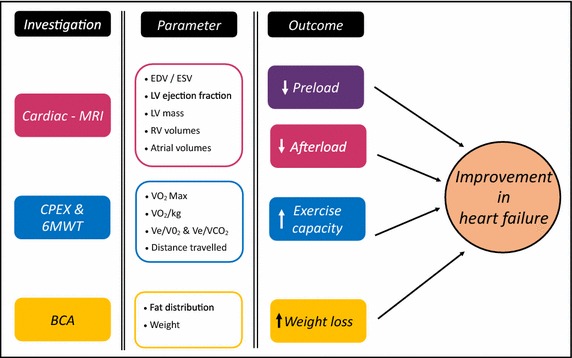
REFORM trial hypothesis. The figure above explains the hypothesis of the REFORM trial where reduction in pre and afterload as well as improvement in exercise capacity and weight loss will all contribute to improvement in heart failure. These features will be measured by cardiac MRI, CPEX, 6MWT and BCA to determine their exact contribution to cardiac function. *CPEX* cardio-pulmonary exercise test; *6MWT* 6 min walk test; *BCA* body composition analysis; *V02* Max Maximum oxygen consumption; *Ve* minute ventilation; *VC02* carbon dioxide production

In this study we propose that there may be unique features of SGLT2 inhibitors that result in a number of haemodynamic and metabolic effects that can ultimately improve survival of patients with HF and DM (Fig. 3).

The primary haemodynamic effect of SGLT2 inhibitors is osmotic diuresis. Approximately 375 ml of extra urine per day is produced in diabetic patients treated with dapagliflozin [24]. Empagliflozin has also been shown to modestly increase urinary volumes among patients with type 1 DM and hyperfiltrating kidneys [37]. The key question is whether or not SGLT2 inhibitors will maintain their diuretic properties in HF patients who are already on loop diuretic therapy and have impaired renal function. A recent meta-analysis of 5 clinical trials showed dapagliflozin 10 mg produced clinically meaningful reductions in HbA1c, weight and systolic BP in HF patients over a 1 year follow up period [38]. Additionally, SGLT2 inhibitors also reduce blood pressure by reducing arterial stiffness [29], and indirectly, as a result of increased diuresis [39, 40]. These effects on intravascular volume and blood pressure will result in reduced preload and afterload respectively, thereby facilitating the positive remodelling of the heart [41, 42].

This effect of SGLT2 inhibitors on positive LV remodelling will have major implications on morbidity and mortality in patients with DM and HF. Indeed, a drug’s effect on LV remodelling is the best surrogate marker of its efficacy and its impact on hard outcomes like survival and disease progression. A consensus paper by Cohn et al. [43] recommends new drug treatment in HF be assessed by their effect on LV remodelling. To emphasise this, Kramer et al. [35] performed a meta-analysis of 30 mortality trials (involving 25 drugs or device interventions) and 88 remodelling studies and showed excellent correlations between effects on LV remodelling and its impact on mortality. Indeed pre-clinical work has shown that SGLT2 inhibitors are capable of reducing LV mass and volume in a rat model with progressive HF [44]. Accordingly, we have selected cardiac MRI measurements of LV EDV and LV ESV as the primary outcome measures for the REFORM Trial. Other measures of LV remodelling such as LV mass and LVEF are key secondary outcome measures. By ensuring the trial is adequately powered for all three facets of LV remodelling we will, for the first time in humans, determine if treatment with an SGLT2 inhibitor be able to induce positive LV remodelling in diabetic patients with HF.

The metabolic effects of SGLT2 inhibitors include weight loss and reduced insulin resistance. Weight loss addresses a universal problem in the HF population; poor effort tolerance, which establishes a vicious cycle that propagates DM and HF. A patient’s weight is a major determinant of their effort tolerance, and physical activity has repeatedly been shown to benefit patients with HF [45–47]. A 24 week study comparing dapagliflozin to placebo showed 2.5–3.5 kg weight reduction as a result of the caloric loss produced by the glycosuria, thus potentially improving overall effort tolerance [48]. Besides weight, the degree of insulin resistance (IR) has also been demonstrated to be inversely related to exercise capacity and directly related to disease severity and clinical outcomes; Doehner et al. had demonstrated that lower insulin sensitivity was associated with significantly lower peak oxygen consumption and LV EF. They also showed reduced insulin sensitivity was an independent predictor of mortality in patients with HF [49]. The relationship between IR and exercise capacity has also been demonstrated in apparently healthy individuals and in diabetic patients [50–52]. The mechanisms for this has yet to be defined, however one possible mechanism could be explained by the endothelial dysfunction caused by the blunting of insulin-induced endothelial nitric oxide synthase (eNOS) as a result of IR. This in turn leads to impaired muscle and cardiac blood flow and glucose transport resulting in reduced exercise capacity [53]. Improving insulin sensitivity has been shown to improve exercise capacity in diabetic individuals with HF [54, 55]. Studies utilising insulin sensitizers such as thiozolidinediones have shown improving insulin sensitivities increases exercise capacity [56]. There is yet to be similar work done around SGLT2 inhibitors, however ZDF rats treated with dapagliflozin [25] and empagliflozin [57] have shown improving insulin sensitivity in treated populations. Also, a randomized double blind placebo controlled trial using dapagliflozin also showed improved insulin sensitivity during hyperinsulinaemic euglycaemic clamping in T2DM patients [58]. By causing weight loss and improving insulin sensitivity, SGLT2 inhibitors could significantly increase the effort tolerance of patients with HF, thereby reducing morbidity and mortality.

The EMPA-REG Outcomes trial revealed potentially beneficial effects of empagliflozin among patients with HF, however it is unknown if these effects are seen throughout the SGLT2 inhibitor class. Other cardiovascular outcome trials such as DECLARE-TIMI 58 (NCT: 01730534) for dapagliflozin and CANVAS (NCT: 01032629) for canagliflozin will reveal whether or not the cardioprotective effects of SGLT2-inhibitor therapy is seen across this drug class. As described above, this study will be able to provide insights into the mechanism of the positive cardiovascular effects conferred by SGLT2 inhibitor therapy and may also help underpin future outcome trials in HF patients involving this drug class.

## Limitations

Firstly this is a relatively small, single centre trial. The use of cardiac MRI has allowed the power of the trial to be preserved despite the small number of participants. However, some differences observed may still be the result of chance and is therefore a limitation of this study. Secondly heart failure is a dynamic disease, as a patient’s intravascular volume changes, their loop diuretic requirement may fluctuate. This may necessitate dose adjustments during the trial which could confound the final outcome. However, every measure is taken to ensure blinding of the investigators is maintained and uniformity in the dose adjustments made.

## Conclusions

HF and DM remain major clinical problems that are associated with increased mortality and morbidity. Therapeutic options to optimize glycaemic control in DM and HF are limited, and only metformin appears to have beneficial effects on CV outcomes. SGLT2 inhibitors could potentially improve LV remodelling and exercise capacity in these patients, thus offering an important new approach to HF management in DM. If they improve exercise capacity and LV remodelling in HF by reducing both preload and afterload, a strong case could be made for a larger trial specifically in HF patients with DM to test if SGLT2 inhibitors really do have a mortality benefit in this unique patient group. Of course these drugs might, conceivably, alter fluid status in HF without altering LV remodelling. We therefore, also need safety data on what (if anything) they do to fluid status in HF patients. If they behave like glitazones, they could increase fluid retention (and by doing so exacerbate HF). On the other hand, since they produce diuresis, they are more likely to have the opposite effect. Only by studying this, will we know the answer. There is currently no data on combining SGLT2 inhibitors and diuretics which is why this combination is not recommended at this point in time. This study will, with careful monitoring, begin to address this combination, and for the reasons given above, it is likely to be more beneficial than harmful in patients with HF. If this study shows that SGLT2 inhibitors have the dual effect of being a diuretic and favourably remodelling the LV in HF patients, then they would stand head and shoulders above other potential second line anti-diabetics for use in HF patients.

## Abbreviations

BCA: body composition analysis
BNP: brain naturetic peptide
BP: blood pressure
CPEX: cardio pulmonary exercise testing
CrCl: creatinine clearance
CRF: case report form
DM: diabetes mellitus
DPPIV: dipeptidyl peptidase inhibitor
EASD: European Association for the Study of Diabetes
EDV: end diastolic volume
EF: ejection fraction
EFSD: European Foundation for the Study of Diabetes
eGFR: estimated glomerular filtration rate
ESV: end systolic volume
FBC: full blood count
GP: general practitioner
HBA1c: glycated haemoglobin
HF: heart failure
IMP: investigational medicinal product
LFT: liver function test
LV: left ventricular
LVEDV: left ventricular end diastolic volume
LVEF: left ventricular ejection fraction
LVESV: left ventricular end systolic volume
LVSD: left ventricular systolic dysfunction
LVH: left ventricular hypertrophy
LVRI: left ventricular remodelling index
MHRA: Medicine and Healthcare products Regulatory Agency
MHFQ: the Minnesota living with heart failure questionnaire
MRI: magnetic resonance imaging
NHS: National Health Service
NYHA: New York Heart Association
QOL: quality of life
RV: right ventricle
RVEDV: right ventricular end diastolic volume
RVEF: right ventricular ejection factor
RVESV: right ventricular end systolic volume
SD: standard deviation
SF-36: short form-36
SGLT2: sodium-glucose co-transporter-2
SPCRN: Scottish Primary Care Research Network
SPSS: statistical package for the social sciences
T2DM: type 2 diabetes mellitus
U&Es: urea & electrolytes
ZDF: Zucker diabetic fatty
6MWT: 6 minute walk test
